# Sex-specific impacts of caffeine on body composition: commentary on a retrospective cohort study

**DOI:** 10.1080/15502783.2025.2454633

**Published:** 2025-01-17

**Authors:** Anna Vittoria Mattioli

**Affiliations:** University of Bologna-Alma Mater Studiorum, Department of Quality of Life Sciences, Bologna, Italy

**Keywords:** Caffeine, free-fat mass index, muscle metabolism, aging, sex differences, estrogen

## Abstract

Background: Understanding the impact of caffeine intake on body composition is a topic of growing research interest. The article “Association Between Caffeine Intake and Fat-Free Mass Index: A Retrospective Cohort Study” by Tian et al. explored this relationship, highlighting a positive correlation between caffeine consumption and fat-free mass index (FFMI). In this letter to the editor, we discuss the broader implications of these findings, emphasizing the need for further exploration of the underlying biological and lifestyle factors influencing caffeine’s effects.

Objective: To provide critical insights into the mechanisms and contextual factors that may explain the observed association between caffeine intake and FFMI, with particular focus on sex-specific differences, hormonal influences, and lifestyle interactions.

Methods: This letter to editor builds on the findings of Tian et al., drawing from related literature and prior research to contextualize the potential mechanisms and broader implications of caffeine’s impact on body composition. The discussion highlights key areas requiring further investigation, including the role of hormonal modulation, genetic variability, and long-term effects on muscle health.

Discussion: The positive association between caffeine intake and FFMI, particularly among women and younger populations, underscores the ergogenic potential of caffeine in enhancing muscle performance and metabolic efficiency. This letter expands on the study by emphasizing the role of hormonal factors, such as estrogen’s modulation of CYP1A2, the liver enzyme critical for caffeine metabolism. The discussion also highlights the complex interplay between caffeine and other lifestyle factors. Finally, this commentary calls attention to the need for more research into the differential effects of caffeine sources, such as energy drinks and supplements, which often include additional ingredients with distinct metabolic and cardiovascular effects. These alternative sources may influence body composition differently than traditional coffee-based caffeine intake, an area that remains underexplored.

## 1. Letter to the editor

### 1.1. To the editor

I recently had the pleasure of reading the article titled *“Association Between Caffeine Intake and Fat-Free Mass Index: A Retrospective Cohort Study”* by Tian et al., which offers compelling insights into the relationship between caffeine consumption and body composition metrics. [1] The use of a robust NHANES database to explore fat-free mass index (FFMI) as an alternative to traditional body mass index (BMI) provides a fresh perspective on understanding dietary and lifestyle impacts on physical health [1].

The study’s findings – that caffeine intake positively correlates with FFMI, particularly among women and younger populations – highlight the potential ergogenic benefits of caffeine [2]. This aligns with existing literature suggesting caffeine’s role in enhancing muscle performance and metabolic efficiency [3]. The observed sex-specific differences warrant further exploration. In our prior works, we underlined that caffeine consumption among women significantly impacted metabolic efficiency and lean mass development, further supporting the findings presented in this study [3,4].

As the authors noted, differences in caffeine metabolism, shaped by hormonal levels and genetic predispositions, likely contribute to the observed variations in its effects. For instance, estrogen plays a critical role in modulating caffeine metabolism by influencing the activity of CYP1A2, the liver enzyme responsible for caffeine clearance [5,6]. These hormonal interactions are particularly significant in populations undergoing hormonal transitions, such as postmenopausal women, and offer intriguing avenues for further research into personalized dietary recommendations and clinical guidelines. The role of CYP1A2 activity in mediating caffeine’s diverse physiological impacts also warrants exploration, as genetic polymorphisms in this enzyme may account for individual differences in caffeine tolerance and efficacy.

One critical area for further investigation is the long-term impact of caffeine on muscle synthesis versus degradation, especially in older adults. Age-related declines in muscle mass, known as sarcopenia, are influenced by metabolic changes that may attenuate caffeine’s anabolic benefits. Understanding how caffeine interacts with these processes could lead to targeted interventions to preserve muscle health and functionality in aging populations [5,7].

Moreover, caffeine’s interaction with other lifestyle factors, such as alcohol consumption and physical activity, adds a layer of complexity to its effects on body composition. Alcohol, for instance, can influence hydration levels, metabolic pathways, and nutrient absorption, potentially modifying caffeine’s impacts. Similarly, physical activity not only amplifies caffeine’s performance-enhancing properties but may also interact with its effects on muscle metabolism and fat oxidation. Investigating these multifactorial relationships is essential to developing a holistic understanding of caffeine’s role in health and disease prevention [5].

Additionally, while this study focused on caffeine intake from coffee, the increasing popularity of alternative sources such as energy drinks and supplements needs further examination. The differential impact of these sources, which often contain additional ingredients influencing metabolism and cardiovascular function, could yield critical insights [8,9].

This study is a valuable contribution to understanding how dietary components influence body composition. It invites further research into the complex interactions of caffeine with genetic, hormonal, and lifestyle factors. The manuscript also underscores the importance of considering sex differences in metabolic and physiological responses.
